# A New Electrical Instrument

**Published:** 1912-02-10

**Authors:** 


### A NEW ELECTRICAL INSTRUMENT.

### The German Invention for Utilising High-frequency Currents.

A recent invention by Nagelschmidt of Berlin is described from German sources as a really great advance in physical therapy. The instrument is somewhat complicated in details, but in principle it is an arrangement whereby power from the mains is transformed into a high-frequency current at about 500 to 1,000 volts. The machine itself is handy and hardly larger than a coal scuttle; and the system of indicators and switches renders it perfectly safe and easy for any one to handle it without any risk.

The once-vaunted high-frequency current has now long ago proved disappointing. This new utilisation of it does not aim at producing therapeutic effects by the mere passage of an electrical current; but at the production of heat by the simple process of using the selected region of the body as a resistance in a circuit in which a considerable current is flowing. Hitherto the strength of electrical currents for use upon patients has been limited; as a rule a few milliamperes alone could be safely allowed. The new apparatus permits currents of ½, 1, or even 2 amperes to be used with perfect safety and without discomfort. The result of these large currents, and of the very considerable resistance to their passage offered by the human body, is the production of heat actually within the tissues; and it is the heat rather than the electricity to which the beneficial results are ascribed.

By a number of experiments it can be demonstrated, not only that heat is developed but that its location is under the most absolute control. To begin with, one has only to grasp the terminals as the current is gradually turned on to realise at once how quickly heat is generated. There is a tingling sensation in each hand, and this is shortly superseded by a pleasant warmth in the hands, wrists and forearms. This warmth can be felt to be in the substance of the limb, not merely on the skin surface; and it can be appreciated by bystanders, who feel the increased heat on placing a finger on the wrist or arm of the patient. When it is desired to apply heat to some peripheral part, such as the knee-joint for instance, it suffices to place a flat electrode on each side of the joint and turn on the current for ten minutes or a quarter of an hour. Osteoarthritic and many other chronic inflammatory affections can thus be treated with, it is said, great success.

### Its Value in Surgical Practice.

There are, however, further applications of the principle which may prove useful in surgical practice. Thus it is easily proved that if the current be made to flow between a broad flat electrode of considerable surface area, and a small-ended one such as the tip of an ordinary probe, the resulting heat is much more intense close to the small electrode than elsewhere, and is in fact sufficient to exercise a powerfully caustic action. To demonstrate this a piece of raw beef can be laid on a broad plate electrode; if a small electrode be then applied to the upper surface of the beef, in a short time a little patch just under it will be quite cooked, whereas the rest is still raw and even cold.

### The Experience of Berlin.

The application of this to surgery may prove of great value. Thus it is suggested, and has in fact been practised in Berlin, to treat an inoperable carcinoma of the uterine cervix with a small pencil-shaped electrode, the other terminal being a broad plate applied to the small of the back. Thus a little of the friable cancerous tissue can be coagulated and destroyed; then this is scraped away with a spoon, and the process repeated. The advantage of thus being able to scrape away a malignant growth without causing hæmorrhage are obvious. Nor is it only inoperable growths that can be dealt with; for it is also proposed that a similar cauterisation should be the preliminary to a Wertheim's hysterectomy, since thus an additional safeguard against the dissemination of cancer cells is created. Then, again, as a means of allaying spasm, as for example in renal or biliary colic and many analogous conditions, this electrically produced heat offers great advantages over the conventional poultice. For rodent ulcer, lupus, and fungating or ulcerated growths of any kind the possibilities of this new treatment are evidently great; and if the method fulfils the hopes which have been formed of it, hospital electrical departments will have to acquire this new and somewhat expensive machine.

